# Explaining and Visualizing Embeddings of One-Dimensional Convolutional Models in Human Activity Recognition Tasks

**DOI:** 10.3390/s23094409

**Published:** 2023-04-30

**Authors:** Gustavo Aquino, Marly Guimarães Fernandes Costa, Cícero Ferreira Fernandes Costa Filho

**Affiliations:** R&D Center in Electronic and Information Technology, Federal University of Amazonas, Manaus 69077-000, Brazil

**Keywords:** human activity recognition, accelerometer data, deep learning, one-dimensional convolutional neural networks, embeddings, explainable artificial intelligence, embeddings visualization, t-SNE, visualization

## Abstract

Human Activity Recognition (HAR) is a complex problem in deep learning, and One-Dimensional Convolutional Neural Networks (1D CNNs) have emerged as a popular approach for addressing it. These networks efficiently learn features from data that can be utilized to classify human activities with high performance. However, understanding and explaining the features learned by these networks remains a challenge. This paper presents a novel eXplainable Artificial Intelligence (XAI) method for generating visual explanations of features learned by one-dimensional CNNs in its training process, utilizing t-Distributed Stochastic Neighbor Embedding (t-SNE). By applying this method, we provide insights into the decision-making process through visualizing the information obtained from the model’s deepest layer before classification. Our results demonstrate that the learned features from one dataset can be applied to differentiate human activities in other datasets. Our trained networks achieved high performance on two public databases, with 0.98 accuracy on the SHO dataset and 0.93 accuracy on the HAPT dataset. The visualization method proposed in this work offers a powerful means to detect bias issues or explain incorrect predictions. This work introduces a new type of XAI application, enhancing the reliability and practicality of CNN models in real-world scenarios.

## 1. Introduction

Artificial Intelligence (AI) refers to the ability of machines to perform tasks that typically require human intelligence, such as visual perception, speech recognition, decision-making, and language translation. AI forms the basis of any technology used for Human Activity Recognition (HAR) [1,2]. HAR systems identify human activities using data captured by wearable devices or smartphones [3,4]. The main goal of HAR is to enhance health outcomes for people who are suffering from chronic diseases, such as diabetes, and Parkinson’s disease, and even for older adults [1,5,6]. HAR has numerous applications in the healthcare and wellness sector for individuals who wish to monitor their health and fitness [3,4].

A variety of sensors can be utilized to implement HAR. One compelling sensor in this domain is the accelerometer. The accelerometer can detect high- and low-frequency movements [3,7]. A gyroscope and a magnetometer can be used to integrate accelerometer data [3,4]. The more information passed to an AI system, the easier it is to learn a pattern. However, in practical applications, more sensors mean more computational resources, and usually they may not be available on a device with limited capacities, such as a smartwatch or smartphone [8]. Therefore, developing a solution that utilizes only one type of sensor renders it more appropriate for use in real-world scenarios.

Traditional Machine Learning (ML) necessitates having an expert to extract valuable information from signal sensors. This would require a significant amount of manual effort [3,4]. Conversely, we have Deep Learning (DL) models that can extract features as good as, if not better than, classical models [3]. A DL model has a large number of hidden layers that enable it to extract high-level features from the data, giving DL models an advantage over conventional machine learning models, which can only learn from hand-crafted features. However, it remains uncertain as to whether the learned features of DL models have the same level of generalizability as hand-crafted features, or if they are dataset-specific.

Convolutional Neural Networks (CNNs) are among the most widely utilized DL models and have emerged as the state-of-the-art method for numerous tasks. In recent years, One-Dimensional Convolutional neural networks (1D CNNs) have been effective in classifying human activity from wearable sensors, such as accelerometers and gyroscopes [3,4,7,8,9]. One-dimensional CNNs are similar to standard convolutional neural networks, but instead of processing two-dimensional images, they process one-dimensional signals, such as time series data. One-dimensional CNNs can be trained end-to-end to extract features directly from raw data.

AI systems are now making significant decisions on our behalf, including influencing criminal sentencing and curating online content [10,11]. However, without understanding why these systems make certain decisions, people are reluctant to trust and rely on such systems. The term “eXplainable AI” (XAI) is used to describe an artificial intelligence system that can provide a human with an explanation of how it reached its decisions [11]. The main issue with current deep learning models is that they cannot provide explanations for their decisions, making them difficult to trust. Novel techniques, such as Gradient-weighted Class Activation Mapping (grad-CAM), are being developed to improve the interpretability of deep models [8,12].

T-Distributed Stochastic Neighbor Embedding (t-SNE) and Principal Component Analysis (PCA) are machine learning techniques for visualizing high-dimensional data [13,14,15]. They possess the capability to condense a set of variables into a smaller set of information, while striving to retain as much input information as possible. These methods are often utilized with raw signals, such as images or time series data. However, the potential for combining these techniques with a deep network has yet to be explored.

This paper describes a method for generating visual explanations of features learned by CNNs applied to HAR. By integrating the training model’s learned features with the t-SNE technique, we offer several explanations of the model’s decision-making process. Moreover, we demonstrate the transferability of the learned features from one dataset to another through the proposed 2D visualization. To the best of our knowledge, this is the first study to present an approach for accurately visualizing learned features in 1D CNNs applied to HAR tasks.

The remainder of this paper is organized as follows. Section 2 introduces the standard protocol used in developing activity recognition applications. Section 3 discusses related works that have utilized the SHO or HAPT databases, the most commonly used XAI methods in HAR, and how t-SNE can be applied. Section 4 describes the methodology, including 1D CNN architectures, the proposed framework, and databases employed. Section 5 presents the metric results achieved for each experiment performed with the SHO and HAPT datasets. Section 6 covers the explainable results. Finally, in Section 7, we conclude our work, presenting some limitations of this study.

## 2. Activity Recognition Protocol

**Figure 1:**
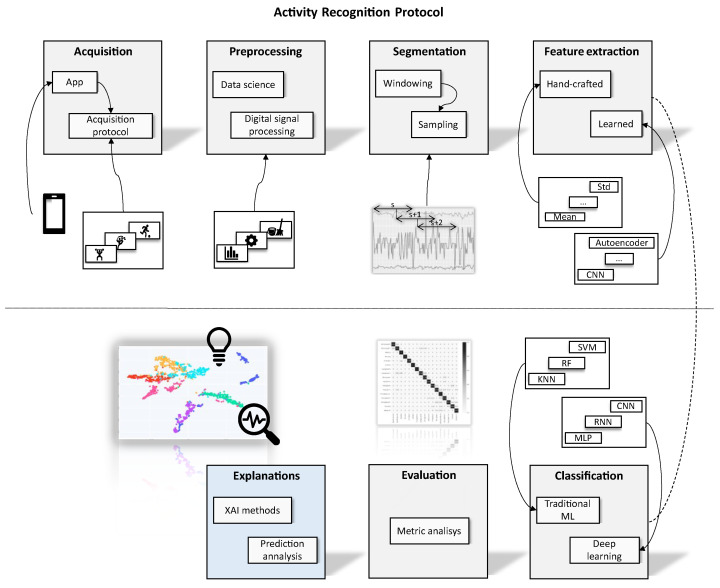
The proposed Activity Recognition Protocol with Explanations includes an additional step for explainability, allowing for analysis beyond metric evaluation. The Classification step utilizes various machine learning algorithms, including Support Vector Machine (SVM), Random Forest (RF), K-nearest neighbors (KNN), Recurrent Neural Network (RNN) and Multilayer Perceptron (MLP).

Most HAR applications utilize the standard Activity Recognition Protocol (ARP) [3,4,16]. However, the activity recognition protocol on HAR applications may differ depending on the specific problem. Usually, ARP consists of six steps: data acquisition, pre-processing, segmentation, feature extraction, and classification and evaluation. We propose a seven-step approach that incorporates explainability. Figure 1 illustrates our proposed protocol.

### 2.1. Acquisition

In the acquisition step of HAR, data is obtained from sensors via an application that adheres to established acquisition protocols. Some studies also employ a camera or microphone for labeling data [17,18]. Accelerometers are the most frequently utilized sensors in HAR and can measure movement in three directions over time, with a sampling rate of 50 Hz being widely used in literature [3]. Accelerometers can detect diverse activities, including static postures, such as sitting and standing, dynamic activities, such as walking, running, and climbing stairs, and transitional activities, such as standing up, sitting down, and lying down [2,3,4].

### 2.2. Preprocessing

Preprocessing in HAR can be performed either during or after acquisition, but it is more common to perform it post-acquisition due to its potential impact on model performance [3]. While sensors may apply basic preprocessing at the hardware level, further preprocessing using digital signal processing, ML, and data science techniques can enhance data quality, identify outliers, and remove noise acquisition [8].

### 2.3. Segmentation

A robust dataset is crucial for developing a successful AI model, and effective segmentation and labeling of samples are essential [8]. Dataset collection protocols involve continuous data collection, followed by segmentation and labeling of the raw data into smaller segments called windows [8]. Due to variations in activity types and times, effective segmentation can balance the number of samples per class or subject and increase the total number of dataset samples [18,19]. For HAR datasets, the optimal window size is typically three seconds, and event-defined or sliding windowing approaches can be employed to segment the data [3]. Sliding windowing often employs windows with 50% overlap to ensure consistency between samples [3].

### 2.4. Feature Extraction

Feature extraction is a crucial step in HAR, involving the transformation of raw data into a reduced set of features that can be more easily processed by machine learning algorithms [2,3]. Two approaches to feature extraction are handcrafted and learned features [2,3]. Handcrafted methods employ mathematical relationships determined by subject matter experts, while learned features are obtained through machine learning algorithms and data correlations. Although deep learning is the most frequently utilized approach for feature extraction in others areas, in HAR many works still employ handcrafted features due to the processing limitations of mobile devices. Handcrafted features can be obtained through statistical attributes in the temporal domain, Fourier transform in the frequency domain, and discretization to obtain symbolic features. In contrast, learned features can be extracted using deep nets, such as CNNs or autoencoders [3]. CNNs extract information through the convolution operation, while autoencoders compress data to extract high-level information in their latent space [8]. The learned features in CNNs are the kernel coefficients, while in autoencoders, they are the latent space of the conversation [8].

### 2.5. Classification

Classification systems can be approached through either a model-driven or data-driven paradigm [3]. Model-driven approaches attempt to manually reproduce the functioning of the physical system by employing composition rules that can describe the issue as an equation. Data-driven methods employ ML to discover data correlations, and are more commonly used in HAR [3]. ML and DL algorithms utilize statistical and mathematical methods to develop algorithms capable of solving classification problems. Traditional ML classifiers, such as naive Bayes, support vector machines, and decision trees, require feature extraction, since they cannot receive raw data. In contrast, DL systems can handle both simple and complex tasks but require a large amount of data. The algorithm choice can significantly impact performance in classification [8]. Once a classifier architecture is selected, it can be trained in Python using popular frameworks, such as TensorFlow or PyTorch for DL, or Scikit-learn for traditional ML [8,20].

### 2.6. Evaluation

After selecting a classification algorithm, we must choose a validation technique to split our dataset into training and testing subsets. This stage is necessary to determine if the model has learned to generalize the task by evaluating it with both seen and unseen samples. Two validation strategies are Subject-dependent (SD) and Subject-independent (SI) strategies [2,3,8]. The SI strategy does not use end-user data during training. In contrast, the SD approach takes advantage of this information when training. The performance of the created model during training and validation must be evaluated using a set of metrics. Accuracy, recall, precision, and f1-score are the most commonly employed evaluation measures in HAR, as shown in Table 1 [3,8].

True Positive (TP), True Negative (TN), False Positive (FP), and False Negative (FN) are the essential elements of all metrics [8] The concept of TP, TN, FP, and FN are based on binary categorization. With a one-vs-all technique, they can be extended to multiclass classification. In this concept, the target class is considered to be positive, while all other classes are combined into a negative class. The most popular metric, accuracy, reflects the proportion of assertiveness overall. Although accuracy is a straightforward metric to comprehend, it does not reflect the true performance of the classifier when we have a dataset with an imbalance of the classes. The precision metric illustrates how the algorithm manages accuracy when predicting positive samples. If we have great precision, our model recognizes TP samples quite effectively. Recall measures how effectively our model deals with false negatives. When there is a strong concern with FN, this metric is applied. Precision and recall are used to calculate the F1-score. When there is an unbalanced distribution of classes, this measure is crucial.

### 2.7. Explainability

To better understand a model’s decision-making process, we can employ XAI methods. Relying solely on numerical results is not the most effective way to determine the best model [12]. Two models with similar results may have learned completely different features and perform in vastly different ways. Moreover, a model with high performance may still have learned a bias from the dataset [8,12]. Therefore, it is important to use XAI techniques to gain insight into how the model makes decisions and to ensure that the model’s predictions are not based on biased or irrelevant features.

There are two distinct approaches on XAI: transparent and post-hoc [11]. Transparent approaches refer to models whose inner architectures and decision-making processes are straightforward and easily understood. Examples of transparent models include the Bayesian model, decision trees, linear regression, and fuzzy inference systems. Transparent approaches are beneficial when internal feature correlations are neither particularly complex nor linear. Post-hoc explainability approaches can reveal the inner workings and decision logic of a trained AI model, providing feature significance scores, rule sets, heat maps, or plain language to assist users in understanding the most relevant information and potential biases [11,21]. In such cases, the post-hoc technique can provide a useful tool for explaining what the model has learned, especially when the relationship between the data and features is not simply a direct one [11].

Within post-hoc methods, we have two distinct approaches: model-agnostic and model-specific [11]. Model-specific approaches provide explainability limitations regarding the learning algorithm and internal structure of a given deep learning model. In contrast, model-agnostic approaches analyze model inputs and predictions in pairs to comprehend the learning mechanisms and generate explanations.

Model-agnostic approaches, such as Local Interpretable Model-agnostic Explanations (LIME) and SHapley additive explanations (SHAP) can be used with handcrafted features and classical ML to determine the importance of input features [22,23,24].

Model-specific approaches are used in Explainable AI (XAI) to explain the decisions made by a specific machine learning model. These approaches are tailored to the model’s architecture, parameters, and training process, and they often rely on knowledge of the internal workings of the model. Examples of model-specific approaches include decision tree induction, rule extraction, gradient-based methods, and layer-wise relevance propagation [12,25,26,27].

These approaches provide valuable insights into the model’s decision-making process and can help improve the model’s performance and reduce biases.

## 3. Related Works

This section discusses related studies in the HAR area, focusing on those that use SHO or HAPT datasets. Next, we discuss the studies that used some XAI techniques in the HAR area. Finally, we discuss the t-SNE technique and applications.

### 3.1. Related Studies in HAR

The earliest implementation of HAR dates back to the 1990s. Since then, numerous strategies and frameworks have been proposed. HAR may utilize different sensors, including cameras, piezoelectric, GPS, microphones, and inertial measurement units. Moreover, several publicly available datasets, including HAPT, UniMiB-SHAR, SHO, and WISDM [3,17,18,28], have been developed to aid research in this field.

CNN architectures are frequently employed in HAR due to their scalability and transition invariance, which contribute to their robustness. Most CNN designs are encoder-like, causing the data to lose dimensionality as it progresses through the layers, leading to the acquisition of advanced semantic meanings. These structures can extract representative features, as well as incorporate handcrafted approaches [29]. Multidimensional data can serve as inputs, as in the case of a nine-dimensional signal composed of a tri-axial accelerometer, a tri-axial gyroscope, and a tri-axial magnetometer. CNNs are not limited to raw data. They can also receive manually created features or features learned from other deep networks. HAR-Net, for instance, is a CNN architecture developed by Dong and Han [30], capable of receiving customized input characteristics. Juefei-Xu et al. [31] calculated the vector sum of accelerometer signals in the x, y, and z axes to create a magnitude signal, which they inputted into a CNN, resulting in superior performance compared to a random forest approach. Ronao and Cho [32] proposed a CNN architecture that employed six-dimensional data as input, consisting of a tri-axial accelerometer and a tri-axial gyroscope, achieving an accuracy of 0.94.

Table 2 demonstrates the frequent utilizations of the SHO and HAPT databases in recent papers. Research on the usage of the HAPT database reveals that some authors choose to group the Postural Transition (PT) classes from the dataset. This grouping strategy may involve dividing the PTs into a single subgroup [18,33] or two subgroups [33,34]. However, there are only a limited number of studies that attempt to classify the PTs into 12 distinct classes.

Regarding works that have utilized the HAPT database, Reyes-Ortiz et al. [18] introduced it by combining an existing database primarily consisting of positional transitions. The authors developed a system that employs a support vector machine classifier and a set of heuristic rules to classify six types of human activities. In their article, they grouped all postural transition activities into a single class, demonstrating that this strategy improved recognition performance of the six Activities of Daily Living (ADLs) considered. Thu and Han [34] created the HiHAR framework, which employs two deep architectures, Convolutional Neural Networks (CNNs) and Bidirectional Long Short-Term Memory Networks (BiLSTM), to classify PT activities into two subgroups [33]. Thu and Han [33] evaluated their architectures considering PTs as a subgroup, two subgroups, and as separate activities.

In their work on the SHO dataset, Jiang and Yin [35] proposed a framework that utilized input from accelerometer, gyroscope, and linear acceleration sensors. The temporal data was converted into an image through a process that utilized the Discrete Fourier Transform (DFT). The framework employed the eight activities available in SHO, but only utilized data from a sensor placed on the wrist. On the other hand, [16] considered all available sensor positions, but only six activities. The activities were selected to be compatible with the physical activities used in other datasets in their study.

### 3.2. Explainable AI with Timeseries

In recent years, AI has been widely adopted across industries and applications. As a result, there is a growing demand for XAI systems that can offer transparent explanations for their decisions and actions. This is especially true in the analysis of time-series data, where interpreting complex patterns and trends over time can be challenging [36,37]. In addition, time-series data is frequently obtained from multiple sensors, generating massive amounts of information that can be difficult to interpret without the appropriate context [37].

In time-series data, XAI techniques can be particularly valuable for understanding complex patterns and trends that evolve over time. Gradient, structure, and surrogate techniques offer different approaches to generating attributions, which can help in explaining AI model decisions [21,38]. Gradient methods are often an efficient starting point for generating attributions, given their computational speed for single samples. On the other hand, structure methods use the learned network weights and biases to propagate a score from the output to the input. Finally, surrogate and sampling methods, such as Local Interpretable Model-agnostic Explanations (LIME) and Shapley Additive Explanations (SHAP), create perturbed instances of the input sample and train an interpretable or game-theoretical model to generate attribution scores.

Assaf and Schumann [39] present a dense-pixel visualization technique for time series data by displaying each time point as a rectangle with the relevance score represented by color. This approach allows for investigating patterns and explaining complex model decisions. However, using grad-CAM [12] for attributions leads to some patterns which are difficult to understand, even with domain expertise. [36] modify this approach by changing the color scale and replacing grad-CAM [12] with a CAM-like algorithm that focuses more on neural network architecture, resulting in improved attributions. Despite these improvements, the visualization still needs to provide insights into the model. Furthermore, it can be challenging to interpret, due to the selected color scale.

Schlegel and Keim [38] combine the approaches of Assaf and Schumann [39] and Viton et al. [36] by overlaying a line plot of the time series data on a background heatmap representing attributions. This approach aims to include both time series and attribution data. Still, it can be more difficult to understand, especially for non-experts.

### 3.3. Explainable AI in HAR

Explainable AI (XAI) refers to a model’s capacity to provide justifications or explanations for its predictions. The majority of XAI techniques [40,41] have been presented for video-based activity recognition. However, generating meaningful explanations for predictions derived from sensor data is more complicated. Therefore, XAI in the context of being HAR sensor-based would involve understanding how the model identifies diverse activities from sensor data and what specific features or patterns it employs to make predictions. This can help enhance the model’s interpretability and transparency and identify and correct any potential biases or errors in the model’s predictions.

Existing research efforts on explainable techniques for sensor-based activity identification are limited, and they examine only intrinsically interpretable models [42]. Bettini et al. [43] claim that, despite the high performance of existing methods for classifying human activities, it is difficult to find a rational explanation of what the model considers to make predictions. Referring to the term XAI, they proposed a framework named XAR, with the letters AR referring to activity recognition. The entire framework was focused on interpretable models, and, in this case, random forest and support vector machines were utilized. They generated explanations by combining the feature values with the feature importance derived from the classifier’s training. Their analysis of a real-world ADL dataset demonstrated that the method successfully provides explanations consistent with conventional wisdom. Furthermore, the proposed framework enables non-expert users with its intuitive and easily understandable explanations of detected movement or activity through natural language.

The authors in [44] utilized a classifier based on rules. In the training step, the model acquires a set of rules that encapsulate the correlations between sensor events and actions and are legible by humans. The findings suggested that the proposed model achieves recognition rates comparable to those of established interpretable classifiers (e.g., Decision Tree, JRip), while producing considerably less complicated rules. In their study, Arrotta et al. [42] introduced DeXAR, an advanced XAI framework that aims to improve the detection of ADLs using DL algorithms. The framework utilizes sensor data and transforms it into semantic images, enabling 2D XAI methods for ADL detection. Furthermore, the authors implemented various XAI algorithms for deep learning, providing non-expert users with natural language explanations based on the resulting heat maps. Finally, to evaluate the effectiveness of the proposed XAI methods, they conducted a thorough evaluation of two different datasets, using both traditional common-knowledge evaluation and user-based evaluation methods.

The authors in [8] proposed employing gradient-weighted class activation mapping (grad-CAM) to generate visual explanations for the decision-making process of deep learning models utilized for HAR and biometric user identification tasks, using accelerometer data. The study found that the high performance of HAR using SD validation was not solely based on physical activity learning but also on learning an individual-specific signature. Overall, the paper suggests that combining explainable techniques with deep learning can facilitate the design of better models, while mitigating overestimation of results. These models can provide a more coherent interpretation of the model’s decision-making processes and can be easily visualized to help understand how the model operates.

This paper presents a novel approach to activity recognition by leveraging the capabilities of XAI techniques. Specifically, we propose a t-SNE-based visualization technique to access the quality of features learned by deep learning models and to identify confusion and misclassification between samples. This approach offers an in-depth understanding of the generalization capabilities of DL models. It has the potential to assist AI researchers in developing more effective models and in understanding their limitations in real-world scenarios.

### 3.4. T-Distributed Stochastic Neighborhood Embedding

T-distributed Stochastic Neighbor Embedding (t-SNE) is a dimensionality reduction technique that performs well for visualization, allowing a projection of the data in low-dimensional spaces that make it simple to apply to very large datasets [13,14]. Using t-SNE during the learning process reduces the dimensionality of the dataset, while preserving its topology, by improving clustering accuracy.

The t-SNE technique utilizes Stochastic Neighbor Embedding (SNE) [14]. The method utilized by both approaches converts high-dimensional data into a probability distribution. Then, it maps this distribution onto a low-dimensional space, such as a 2D plane or a 3D space. Principally, the SNE converts high-dimensional Euclidean distances between data points into conditional probabilities that represent similarities. The t-SNE technique utilizes the student t-distribution with one degree of freedom as the heavy-tailed distribution in the low-dimensional map.

The t-SNE algorithm begins by calculating the pairwise similarities between all high-dimensional data points [14]. These similarities are represented as a high-dimensional probability distribution, a probability distribution with a high number of dimensions. Then, t-SNE generates a low-dimensional probability distribution by assigning a probability to each pair of points in the low-dimensional space. This low-dimensional probability distribution is intended to closely resemble its high-dimensional counterpart.

Kullback–Leibler divergence is the t-SNE cost function, which measures the difference between the high-dimensional and low-dimensional probability distributions [14]. The cost function is defined as:

(1) $$C_{t-\mathrm{SNE}}=\mathrm{KL}\left(P||Q\right)=\sum_{i}\sum_{j}p_{ij}\log\frac{p_{ij}}{q_{ij}}$$

Kullback–Leibler divergence (KL) is a measure of the difference between two probability distributions. In the t-SNE algorithm, KL  divergence is used as the cost function, where *P* and *Q* represent the high-dimensional and low-dimensional probability distributions, respectively. In Equation (1), $p_{ij}$  denotes the probability that the high-dimensional point *i* is selected as the neighbor of point *j*, whereas $q_{ij}$  is the probability that the low-dimensional point *i* is selected as the neighbor of point *j*.

Through adjusting the positions of the points in the low-dimensional space, t-SNE seeks to minimize this cost function. The gradient of the cost function with respect to the low-dimensional coordinates is typically calculated and used to update the coordinates using a gradient descent algorithm.

In HAR, t-SNE is usually used to visualize the separation of the data. Dharavath et al. [45] used t-SNE to visualize the data distribution in a HAR dataset. The dataset contained an accelerometer and gyroscope readings while performing activities such as walking, walking upstairs, walking downstairs, standing, and lying. The author ran the raw data through the t-SNE method, comparing the generated visualization with a visualization obtained with the PCA technique. The visualization with t-SNE was significantly better.

Thakur et al. [46] used PCA to extract the characteristics of human activities from both accelerometer and gyroscope sensors. The obtained features were input into t-SNE to visualize the data’s separation. The extracted features from PCA were also fed into an ensemble of three ML classifiers to identify six distinct human physical activities.

## 4. Methodology

This section explains the architectures implemented, the public database used and the proposed framework. In practical applications, more sensors mean more computational resources, which are usually not available on a device with limited capacities, such as a smartwatch or smartphone [8]. Therefore, developing a solution that utilizes only one type of sensor renders it more appropriate for use in real-world scenarios.

### 4.1. Architectures

The simulations were conducted utilizing CNN1 and CNN2 convolutional network architectures, initially introduced by Aquino et al. [8]. The models were trained and implemented using the TensorFlow 2 framework [20]. Initially, we emphasized the commonalities between both designs. In both instances, the Adam optimizer was employed with its default hyperparameters. The models underwent 300 training epochs. Each convolutional block comprised two layers, each of which contained 100 feature maps. The ReLU activation function was utilized. In every max-pooling layer, the pool size and stride were set to 2. For dropout, a value of 0.5 was applied [47]. The Softmax layer contained *n* neurons, where *n* represents the number of classes for the problem. A custom callback was employed to create checkpoints after each training epoch, selecting the optimal model based on the macro F1-score metric for the validation set. The batch size was set to 256. Both architectures received identical 151×3  signals at their inputs.

**Figure 2:**
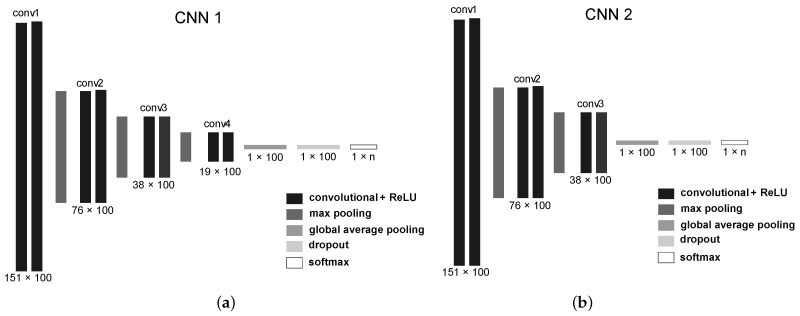
The CNN architectures implemented with TensorFlow 2. (**a**) shows the CNN1 architecture with 4 convolutional blocks. (**b**) shows the CNN2 architecture with 3 convolutional blocks.

The differences between the two architectures are now discussed. The first architecture comprised four convolutional blocks, each with two convolutional layers, while the second architecture incorporated only three convolutional blocks. Furthermore, CNN1’s kernel size was eight, whereas CNN2’s was four. Figure 2 illustrates both architectures.

### 4.2. Databases

This section provides specific information about the datasets used in this study. Human activity recognition (HAR) can employ various sensors, such as accelerometers, gyroscopes, and magnetometers. Accelerometers are highly effective in detecting movements, and many related studies have used this type of sensor alone, due to its relevance and reliability in HAR [3,4]. Although combining sensors can improve accuracy, this may not be feasible for devices with limited processing capacity, such as smartphones or smartwatches [3,8]. Therefore, using a single sensor, such as an accelerometer, is a more practical approach for real-world HAR applications [8,9,48].

#### 4.2.1. SHO

The authors collected data for seven physical activities: walking, running, sitting, standing, jogging, biking, walking upstairs, and walking downstairs [28]. Ten volunteers performed each task for 3–4 min as part of the data capture project. All ten subjects were men between the ages of 25 and 30. Except for cycling, the studies were conducted inside a university facility. Each of these participants was equipped with five smartphones for use in five different body postures: right pocket, left pocket, belt position toward the right leg using a belt clip, right upper arm, right wrist.

For the trials, Samsung Galaxy SII (i9100) smartphones were used. For each movement, data was collected at a rate of 50 samples per second for all five places simultaneously. Accelerometer, gyroscope, magnetometer, and linear acceleration sensor information were collected [28].

In this work, for the dataset generation, a 3-s window was used, with 50% overlapping. The data were collected only from the waist position using a belt, and only the accelerometer sensor was considered. For the subject-dependent (SD) method, we implemented a randomized partitioning strategy with shuffling, adhering to a conventional 70/30 split for training and validation. Additionally, for the subject-independent (SI) approach, we endeavored to maintain the same proportion in the partitioning, yielding 7 subjects for training and 3 subjects for validation. Validation was performed using subjects 1, 2, and 3.

SHO is a high balanced dataset. For our setup, a total of 4130 samples was obtained, with exactly 590 samples per class, and all ten subjects provided the same number of samples.

#### 4.2.2. HAPT

The UCI Human Activity Recognition Using Smartphones Dataset was expanded in this dataset [18]. Instead of the pre-processed signals from the smartphone sensors that were supplied in version 1, this version offered the original unprocessed raw inertial signals from the sensors. Additionally, the activity labels were revised to incorporate postural changes that were absent from the earlier dataset version.

The public HAPT dataset was obtained from thirty participants ranging in age from 19 to 48 years [18]. The dataset contained raw inertial signals obtained from 3-axial linear acceleration and 3-axial angular velocity sensors integrated in a smartphone equipped at the waist by the user. It included 6 basic activities (BAs): standing, sitting, laying, walking, walking upstairs, and walking downstairs#, and 6 postural transitions (PTs) between three static postures, stand-to-sit, sit-to-stand, sit-to-lie, lie-to-sit, stand-to-lie, and lie-to-stand.

The data was collected at a constant sampling frequency of 50 Hz. Signals were then synced with experiment recordings so that they could be used as the ground truth for hand labeling [18].

For dataset generation the same setup used in SHO dataset was applied, namely, a 3-s time window with 50% overlapping, and only an accelerometer sensor. The data were collected only from the waist position using a belt. Table 3 displays more details about the data distribution of the dataset. As shown in the N° Sample column, HAPT was an unbalanced dataset. Finally, as shown in the N° subjects’ column, we can see that not all participants performed all activities, especially for PT.

For the subject-dependent (SD) method, we implemented a randomized partitioning strategy with shuffling, adhering to a conventional 70/30 split for training and validation. In the SI approach, we took into account that not all individuals performed all physical activities. Therefore, for the validation subset, the first nine individuals, who performed all activities, were chosen: subjects 1, 2, 3, 4, 5, 6, 13, 17, and 18. The remaining subjects were used in the training subset.

### 4.3. Proposed Framework

**Figure 3:**
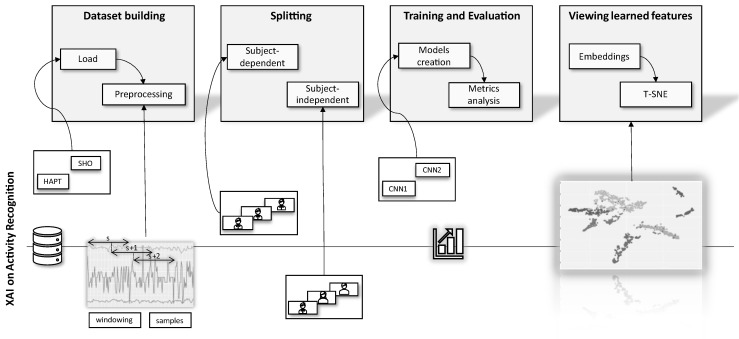
A schematic representation of the proposed framework for XAI on activity recognition. The framework consists of four steps: dataset building, splitting, training and evaluation, and viewing learned features with our proposed t-SNE visualization technique, where each color represents a different activity.

To summarize, we propose the framework illustrated in Figure 3. In the dataset building step, we loaded the SHO and HAPT databases and performed an exploratory analysis of the data.

This exploratory analysis allowed us, for example, to identify that not all individuals performed all activities in the HAPT database. We also defined a window size of 3 s with 50% overlapping. After this, the data was subdivided in the splitting step according to SD or SI validation strategy. Then, the models were implemented and evaluated, with in training and evaluation steps, and their results contrasted using performance metrics. Again, we used the CNN1 and CNN2 architectures. Finally, we conducted an in viewing learned features step, in which we obtained the embeddings for the architectures, and used this as input for the t-SNE. By doing this, we had a visualization that clearly explained how the model performed the data distribution from the learned features.

In this work, we proposed using a framework to visualize the power of features learned from a dataset or how features learned from one dataset behave in another unseen dataset. For evaluation on the same trained dataset we, first, used the dataset to train the two CNN architectures, CNN1 and CNN2. We chose the best architecture based on the achieved numerical results. We obtained XAI visualizations and provided explanations of the decision-making process.

In other words, the HAPT samples were passed through this network, and a vector with information from one layer before classification was obtained. After that, new visualizations were performed, which allowed us to conclude the limitations of the learned features.

The proposed framework allows for the visualization of the effectiveness of features learned within a single dataset and the examination of their performance on an unseen dataset. This is achieved by implementing the proposed activity recognition protocol and including a visual explanation step to gain insight into the ability of learned features to differentiate activities on another dataset.

## 5. Metric Results

### 5.1. SHO

In this study, we evaluated and trained two CNN architectures, CNN1 and CNN2, employing both the SD and SI strategies. To enable a more precise comparison with other relevant studies, we utilized both macro-average and weighted-average metrics. The macro-average metric considers the impact of the metric on each class individually, while the weighted-average metric accounts for the metric’s results on each class, weighted by the number of samples assessed. The macro-average metric is especially beneficial for handling imbalanced systems, as it does not discriminate between classes with larger data [8]. Conversely, the weighted-average metric offers a more comprehensive understanding of the system, assuming that the class distribution in the dataset resembles that of real-world data [8]. Detailed performance results for each network, considering both the SD and SI strategies, are presented in Table 4 and Table 5.

The results obtained from the SD validation strategy were marginally superior to those acquired with the SI strategy for both trained architectures, across all metrics. This outcome was expected based on prior research, which suggests that the SI validation method is more rigorous and offers a more accurate depiction of the network’s performance in real-world scenarios, as the network is not provided with prior knowledge about how individuals perform an activity [4].

The CNN2 architecture outperformed CNN1 using the SD strategy, while, with the SI strategy, CNN1 performed better. The performances of both architectures were closely matched, with a difference of only 0.02% in the SD strategy and 0.3% in the SI strategy.

**Figure 4:**
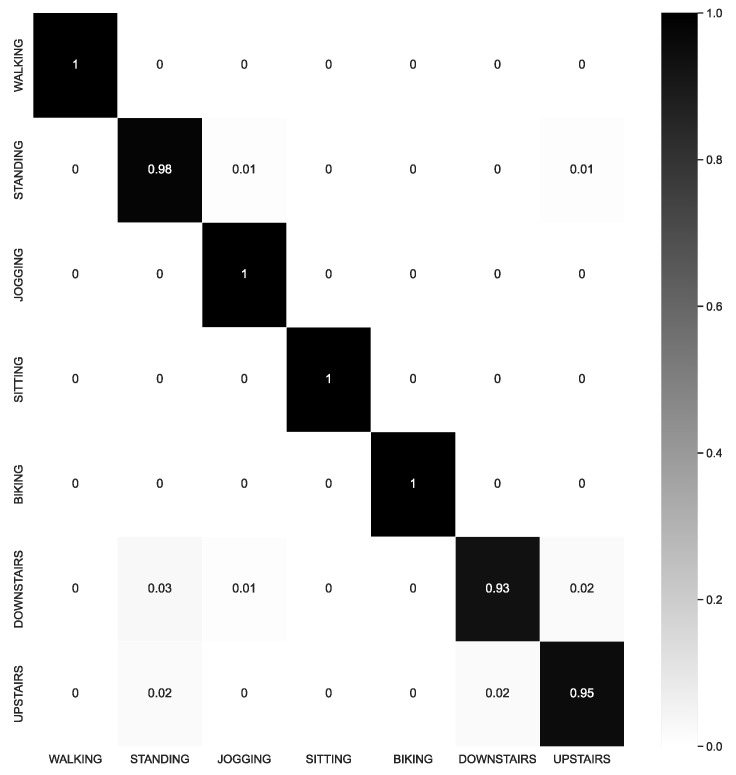
Confusion Matrix of CNN1 with subject-independent, with 3 subjects in validation for all 8 classes in SHO dataset.

The SI approach was determined to be more demanding and better aligned with how the model would be evaluated in real-world scenarios. Consequently, greater emphasis was placed on the SI results. By examining the confusion matrix depicted in Figure 4, we gained insight into the challenges encountered by the CNN1 model when utilizing the SI approach.

### 5.2. HAPT

Since this database was highly imbalanced, the results were anticipated to be less remarkable, given that there were 12 classes and only a few samples of the postural transition activities. The results are displayed in Table 6 and Table 7.

The results obtained between the two approaches were comparable. Considering the macro F1-score, there was a difference of less than 1% between the best architecture using the SI and SD approaches. This difference was under 3% in terms of the accuracy metric. The weighted metrics achieved the best results in both approaches, as anticipated, due to the imbalanced nature of the dataset.

**Figure 5:**
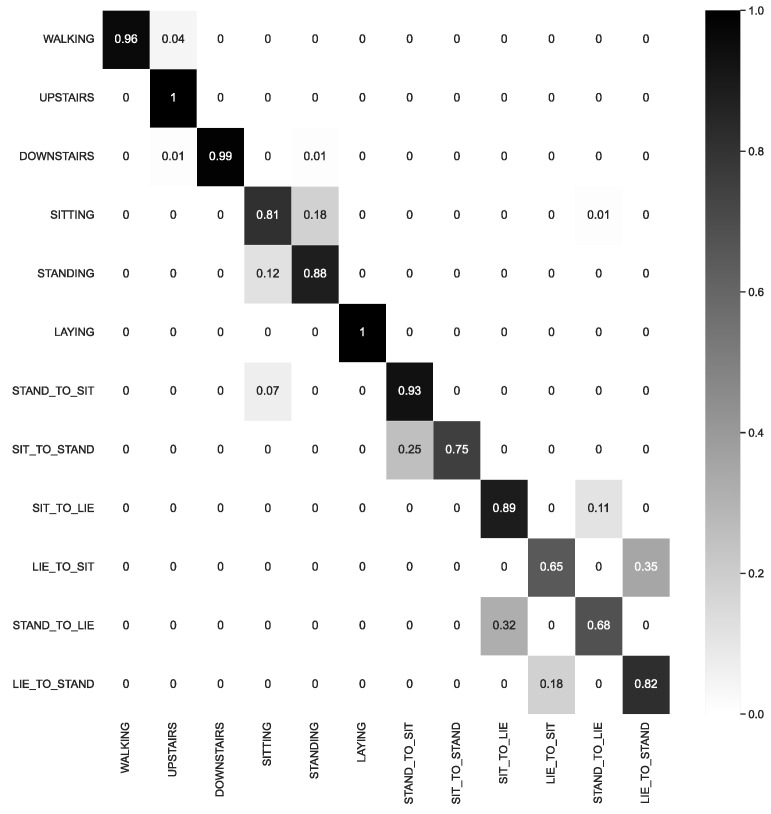
Confusion Matrix of CNN1 for SI approach, with 9 subjects in validation set, using all 12 classes in HAPT dataset.

Figure 5 displays a confusion matrix for the best architecture utilizing the SI approach.

## 6. XAI Results

In the XAI results section, we explore the outcomes obtained with the deep network CNN1 on the SHO and HAPT datasets, employing the proposed t-SNE visualization through the learned features. The results are scrutinized and interpreted to emphasize the efficacy of t-SNE in detecting bias issues and identifying mislabeled samples. The visualization further illustrates the deep network’s generalization capability in distinguishing activities and its constraints when applied to a distinct dataset. Ultimately, the discussion offers insights into how t-SNE visualization can augment the comprehension of deep networks in HAR tasks.

### 6.1. SHO

**Figure 6:**
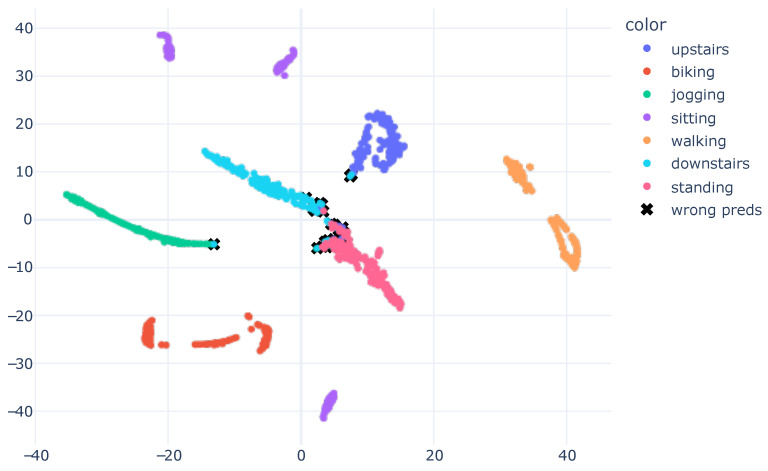
T-SNE Embedding Plot obtained from the CNN1 architecture with HAR using the SI approach for the SHO dataset. We utilized 40 perplexity and conducted 500 iterations.

Following the training of the models and evaluation of the numerical results, we proposed employing XAI to generate visual representations that elucidate the model’s learning process. We extract the model’s embeddings up to one layer before the Softmax, and, subsequently, trained a t-SNE based on this information. The outcome was a two-dimensional embedding that endeavored to preserve the information present in the model’s output. This approach enabled clear visualization of the model’s acquired knowledge regarding data characteristics and how the data was partitioned, based on the learned features. Figure 6 presents the results graph.

The visual results obtained corresponded with the numerical results presented in Table 5, wherein the model achieved exceptional performance with an accuracy of 0.98. In the plot, the data from different classes were dispersed. Confusion occurred between activities, such as downstairs and standing. This outcome was also evident in the confusion matrix shown in Figure 4, where the downstairs class attained lower precision than other activities.

As depicted in Figure 6, black x marks were drawn on samples with incorrect predictions to identify the model’s errors. The resulting representation enabled verification that the incorrect predictions were readily observed and explained. For instance, we examined the misclassified sample within the jogging cluster, indicated by a black x. The ground truth for this sample was the downstairs class. This confusion arose due to the features learned by the model, which positioned this sample near the jogging cluster. This may have occurred as a result of a limitation in the trained model, which might have learned characteristics that did not optimally separate the data. Alternatively, this sample could have been mislabeled. Nonetheless, the obtained visualization allowed for an understanding of why the model made an error.

In the visualization, samples from downstairs were more dispersed, indicating that the downstairs class was the most challenging in the dataset. The model may be more susceptible to predicting this class in real-world scenarios. Moreover, by analyzing the results of the obtained graph, it became evident that there were subgroups within the same class, such as sitting, which featured three distinct subgroups scattered throughout the view. Similarly, even walking had two subgroups. These analyses were not possible solely through numerical results, and the visualization presented various opportunities to explain the model’s predictions.

An intriguing observation was made by analyzing the sitting activity. There were three clusters, which corresponded to the three different subjects present in the validation subset. This finding suggested that the learned features may have the potential to not only distinguish the sitting activity, but also differentiate the subjects.

**Figure 7:**
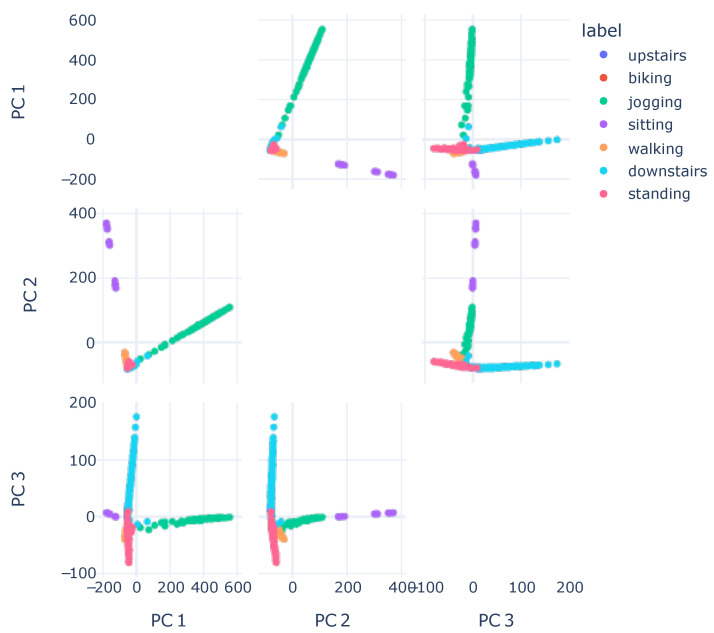
Embeddings visualization with PCA, combining three components, 2 by 2.

To further highlight the capabilities of this visualization, Figure 7 displays a visualization using PCA. The method applied was similar to t-SNE. Initially, we utilized the model’s embeddings output, one layer before the classification, as input for the PCA. Next, we applied PCA to derive only three components, which were employed to generate the plot. We calculated merely three components to demonstrate that there was no combination of components for which the resulting visualization was as informative as the one obtained with t-SNE.

It was anticipated that the data would exhibit clear class separation; however, this visualization did not align with the numerical results. Providing explanations was challenging, since, in this visualization, the majority of classes were situated close to each other. This outcome might have arisen because PCA lacks the capability to extract relevant information from non-linear data, which could have been the case with the features learned by the model.

**Figure 8:**
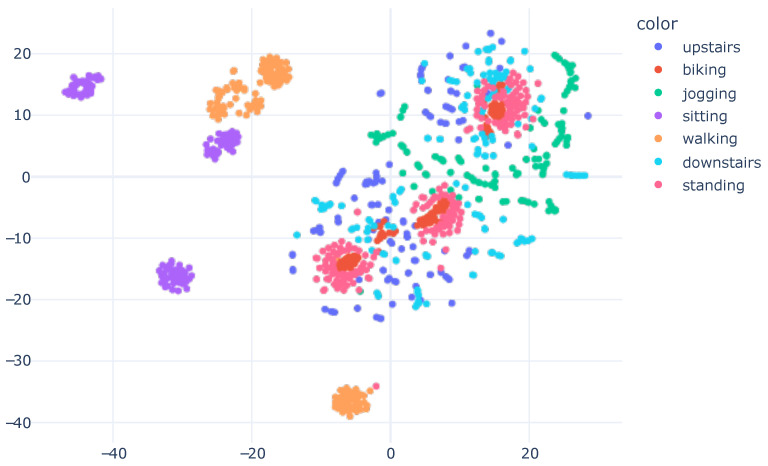
SHO accelerometer raw data visualization with t-SNE, concatenating X, Y and Z information. We utilized 40 perplexity and conducted 500 iterations.

The representation in Figure 6 demonstrates how the model learned to separate the data based on the features it acquired. We plotted the graph displayed in Figure 8 to analyze the distribution of raw accelerometer data. To generate this plot, we concatenated the data from the X, Y, and Z axes of the accelerometer and used it as input for the t-SNE algorithm. Our work introduces the innovation of applying t-SNE one layer before the model’s classification, setting it apart from the standard approach of applying t-SNE on raw data, as seen in previous works [13,14,45,46].

By visualizing the plot of the learned features during the model’s training, it became evident that the model underwent a transformation in regard to the input data. In its original form, the raw data presented a challenge in differentiating between human activities. However, after the model learned and distilled the relevant characteristics, the separation between classes became more evident and distinguishable. This visualization offered valuable insights into the model’s inner workings and assisted in enhancing its performance.

### 6.2. HAPT

**Figure 9:**
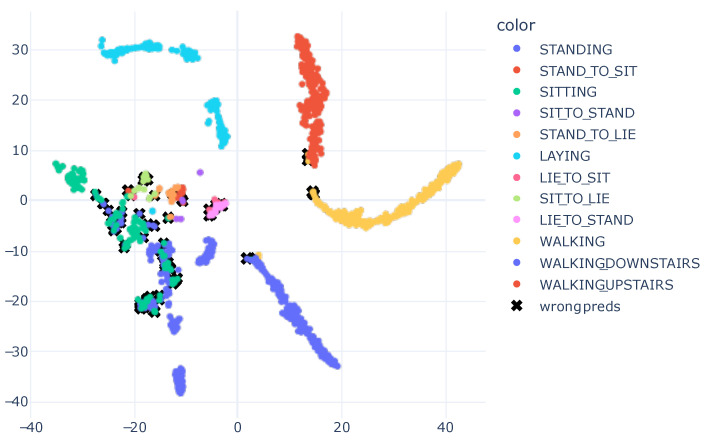
HAPT t-SNE visualization. We utilized 40 perplexity and conducted 500 iterations.

For the HAPT dataset, based on the results presented in Table 7, a more significant confusion between samples was expected compared to the representation obtained with the SHO dataset. Examining Figure 9, it is possible to observe a lesser separation between samples of different classes. The main issue lay in the Postural Transitions (PT) classes. This result was also anticipated when observing the confusion matrix in Figure 5.

**Figure 10:**
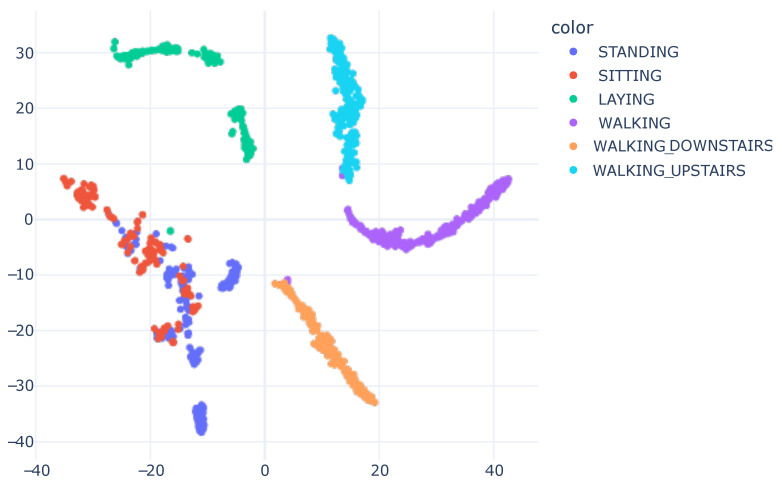
T-SNE Embedding Plot obtained from the CNN1 architecture with HAR using the SI approach for HAPT dataset, excluding PT activities. We utilized 40 perplexity and conducted 500 iterations.

To demonstrate that the model learned relevant features for the other activities, excluding Postural Transitions (PTs), Figure 10 is presented. Other works have already considered these PTs as one or two subgroups. Some studies only use PTs to enhance the performance in recognizing other activities.

The model encountered a familiar challenge, similar to the SHO dataset, with the standing class proving difficult to classify. However, in contrast to the SHO dataset, confusion arose between standing and sitting classes. Further analysis revealed the presence of subgroups within the laying, standing, and sitting classes.

**Figure 11:**
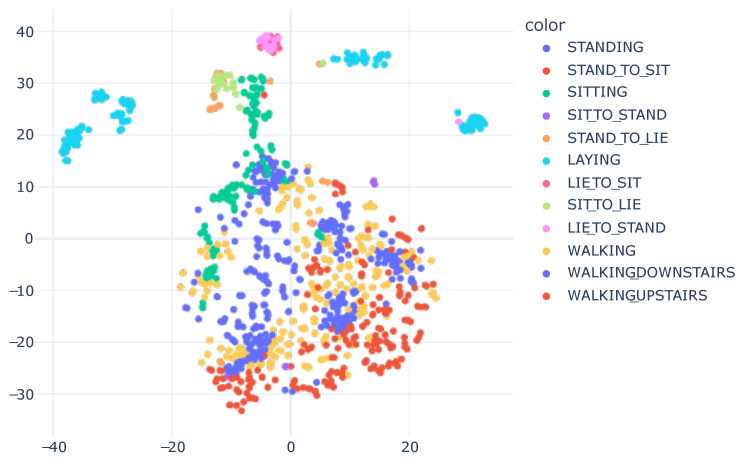
Accelerometer raw data visualization with t-SNE, concatenating X, Y and Z information. We utilized 40 perplexity and conducted 500 iterations.

To better illustrate the original data distribution, Figure 11 displays the raw accelerometer data using t-SNE. The same process utilized to generate Figure 3 was applied to the HAPT dataset.

As occurred with SHO, utilizing raw data as t-SNE input was a challenge because there were no highly relevant features, in contrast to Figure 9, where the features were those learned by the neural network and had mathematical relevance.

Overall, the models demonstrated their ability to learn meaningful features, as evidenced by the t-SNE visualizations and performance metrics obtained on both the SHO and HAPT test sets. Next, we assess whether the features learned in one dataset could accurately classify human activities in another dataset.

### 6.3. SHO Features into HAPT

To analyze the relevance of the features, we used a network trained on the SHO dataset to extract features from the HAPT dataset. To do this, we performed a prediction with the SHO model, propagating the HAPT samples and considering the result before the classification layer. This result was used as input to the t-SNE algorithm.

**Figure 12:**
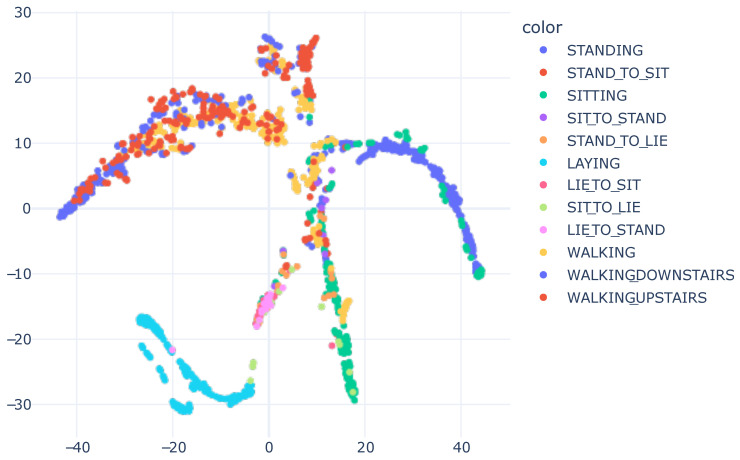
T-SNE Embedding Plot obtained from the CNN1 architecture trained with SI approach with SHO dataset into HAPT dataset. We utilized 40 perplexity and conducted 500 iterations.

Figure 12 shows the resulting representation. However, the SHO dataset’s learned features needed to be universal to be useful for other datasets. In this case, the SHO features caused the pattern of the HAPT dataset to be more confusing than the raw data. Although Figure 6 successfully distinguished between activity types using the SHO dataset as a reference, this approach produced unsatisfactory results when applied to the HAPT dataset.

### 6.4. HAPT Features into SHO

**Figure 13:**
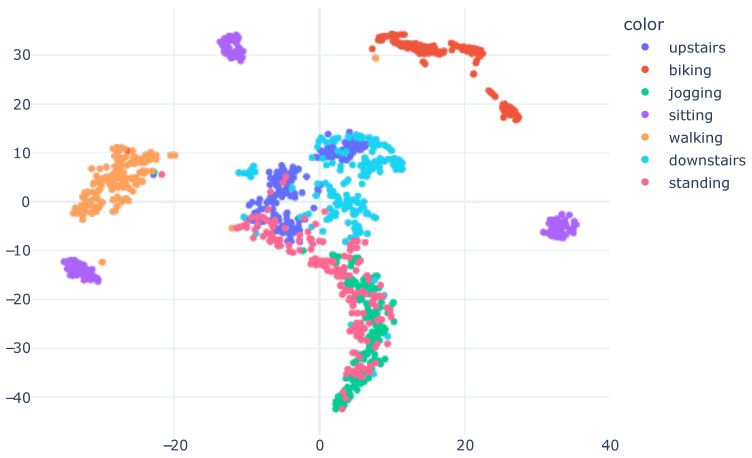
T-SNE Embedding Plot obtained from the CNN1 architecture trained with SI approach with HAPT dataset into SHO dataset. We utilized 40 perplexity and conducted 500 iterations.

To analyze the relevance of the features, we used a network trained on the SHO dataset to extract features from the HAPT dataset, as displayed in Figure 13. To do this, we propagated the HAPT samples through the SHO model and considered the output before the classification layer. We then used this output as input to the t-SNE algorithm.

When examining the HAPT dataset, it became evident that utilizing learned features resulted in a more precise differentiation of the data than using the raw accelerometer data. Figure 11 shows that the contrast between the two approaches was clear. The Sitting, Walking, and Biking classes were well separated, unlike what was observed when analyzing the raw data dispersion, shown in Figure 8, where the Biking class was grouped with other classes. The Upstairs, Downstairs, Standing, and Jogging classes were grouped in close regions, but there was still noticeable separation between the samples of these classes.

## 7. Conclusions

T-SNE is frequently utilized to explore the separation of raw data in a comprehensible manner. By applying this method to the output of a DL model, we introduce a novel post-hoc and model-specific approach to the XAI field. Implementing t-SNE on the output of a DL model generates a visualization that can convey a general understanding of the model’s learned features during the training process. This is distinct from the explanations produced by other established XAI methods, such as decision tree induction, rule extraction, or gradient-based techniques. In contrast to these alternatives, the proposed methodology is adept at presenting a holistic overview of a DL model across a data subset. By enhancing our understanding of the model’s behavior, this technique can function as a valuable instrument for debugging and optimizing the model’s performance.

The t-SNE embedding visualization demonstrated its potential in offering valuable insights. Nonetheless, certain limitations must be acknowledged when interpreting the results of this study. For instance, the performance of this visualization technique with alternative data types, such as electrocardiogram signals or other sensor data, remains to be explored, potentially impacting the generalizability of the approach across different domains.

Moreover, although the visualization proved effective in analyzing the decision-making processes of a CNN-based model on identical or diverse datasets, additional research is warranted to assess its applicability to various DL algorithms. Investigating alternative network architectures, such as recurrent neural networks, ConvLSTM, Bidirectional networks, and hybrid nets, may furnish a more comprehensive understanding of the t-SNE embedding visualization’s versatility with respect to different deep learning models.

Hence, while the present study contributes valuable insights into the prospective utilization of t-SNE embedding visualization for human activity recognition, based on accelerometer data, it is imperative to recognize the limitations of the proposed approach and continue investigating its efficacy across other domains and with different deep learning algorithms.

Upon applying t-SNE to a network trained on a distinct dataset, the resulting data separation appears less distinct. Instead, the classes display a degree of mixing, as evidenced in Figure 12, where the distribution was inferior to that observed when utilizing raw data as input, as depicted in Figure 11. This confusion may arise from the network’s inability to learn pertinent features beyond its original database. Such limitations could stem from discrepancies in data collection, including differences in the individuals involved, the sensors employed, or the collection software implementations. For instance, although both databases position the sensor at the waist using a belt, variations in orientation and characteristics may still arise. If the belt is fastened near the navel, the features obtained may differ from those acquired when the phone is attached closer to the side. The closer the sensor is to the center of mass, the smoother the signal, and this property may influence the neural network’s capacity to extract relevant features.

The features extracted by the model trained on the HAPT dataset proved to be more informative, enabling more accurate discrimination of the data than the raw data itself when applied to the SHO dataset, as can be observed when comparing Figure 11 and Figure 13.

The proposed visualization offers a lucid representation of the data distribution based on the network’s learned features. This aids in accomplishing several objectives, such as detecting and rectifying critical confusion, pinpointing biases, identifying mislabeled samples, revealing potential subgroups within a group, and more.

This approach is not confined to HAR alone. It can be employed in any problem involving time series data and deep learning algorithms, particularly convolutional neural networks.

This study paves the way for various research avenues, including the following:

1. Which validation strategy results in better separation of classes in a HAR dataset, SI or SD?
2. If additional sensor position data were incorporated into the SHO database, would it improve the separation of HAPT classes?
3. Is it possible to reverse the process? Starting from t-SNE embedding coordinates, can raw data be retrieved? If so, could this be utilized for data augmentation in the dataset?
4. How do the embeddings derived from other deep network models, such as LSTM, BiLSTM, GRU, MLP, and hybrid models, perform?
5. How should the proposed visualization technique be employed to avoid class confusions, modifying the architecture to improve and facilitate the differentiation of challenging classes?

## Figures and Tables

**Table 1:** Most commonly used metrics in HAR systems.

Metric	Equation
Accuracy	$\frac{TP+TN}{TP+TN+FP+FN}$
Recall	$\frac{TP}{TP+FN}$
Precision	$\frac{TP}{TP+FP}$
F1-score	$2\times \frac{\left(Precision\times Recall\right)}{Precision+Recall}$

**Table 2:** Performance comparison of different HAR models using the HAPT and SHO datasets; N × M—N is the input size and M is the input dimension; FE—feature extracted; Acc—Accelerometer; Gyro—gyroscope; BA—basic activities; PT—postural transition; SD—subject-dependent; SI—subject-independent; CV—cross-validation; LOSO—Leave-one-subject-out.

Author	Dataset	Model	Input (N × M)	Validation Strategy	Classes	Best Performance
Reyes-Ortiz et al. [18]	TAHAR system with SVM	561 × 1 FE vector (Acc + Gyro)	N/A	No validation subset	6 BA + 1 PT group	0.9700 accuracy
Thu and Han [34]	HAPT	HiHAR-8 and CNN	128 × 6 (Acc + Gyro)	SI: 24/6	6 BA + 2 PT groups	HiHAR-8: 0.9798 accuracy
						CNN: 0.9440 accuracy
Thu and Han [33]	HAPT	BiLSTM * and CNN	No info about input shape. FE: DWT ** (Acc + Gyro)	SD: Hold-out 80/20	6 BA + 1 PT group	BiLSTM: 0.9634 accuracy
					6 BA + 6 PT	BiLSTM: 0.9487 accuracy
						CNN: 0.9171 accuracy
Jiang and Yin [35]	SHO, only wrist sensor position	2D CNN	36 × 68 - image generated from DFT *** (Acc + Gyro + Linear acceleration)	SD: Hold-out 70/30	8 BA	0.9993 accuracy
Braganca et. al. [16]	SHO - all available sensor positions	Random forest	64 × 1 FE (Acc)	SD: 10-CV, SI: LOSO	6 BA	SD: 0.98 MAA ****, SI: 0.8412 MAA

* BiLSTM = Bidirectional Long Short-Term Memory; ** DWT = Discrete Wavelet Transform; *** DFT = Discrete Fourier Transform; **** MAA = Mean Average Accuracy

**Table 3:** Activity and postural transition (PT) classes in the dataset with corresponding labels, number of samples, and number of subjects.

Action	Label	N° Samples	N° Subjects
BA	Standing	855	30
	Sitting	782	30
	Laying	851	30
	Walking	746	30
	Walking Upstairs	687	30
	Walking Downstairs	614	30
PT	Stand-to-sit	39	24
	Sit-to-stand	14	10
	Sit-to-lie	59	30
	Lie-to-sit	51	28
	Stand-to-lie	71	29
	Lie-to-stand	50	29

**Table 4:** Classification performance of CNN1 and CNN2 in HAR, with the SD strategy and 70/30 split on SHO dataset.

Model	Macro Average			Weighted Average			Accuracy
	Precision	Recall	F1-Score	Precision	Recall	F1-Score	
CNN2	0.9976	0.9976	0.9976	0.9976	0.9976	0.9976	0.9976
CNN1	0.9966	0.9969	0.9967	0.9968	0.9968	0.9968	0.9968

**Table 5:** Classification performance of CNN1 and CNN2 in HAR, with the SI strategy and 3 subjects on validation.

Model	Macro Average			Weighted Average			Accuracy
	Precision	Recall	F1-Score	Precision	Recall	F1-Score	
CNN2	0.9816	0.9814	0.9814	0.9816	0.9814	0.9813	0.9814
CNN1	0.9786	0.9782	0.9781	0.9786	0.9782	0.9781	0.9782

**Table 6:** Classification performance of CNN1 and CNN2 in HAR, with the SD strategy and 70/30 split.

Model	Macro Average			Weighted Average			Accuracy
	Precision	Recall	F1-Score	Precision	Recall	F1-Score	
CNN2	0.8724	0.8817	0.8742	0.9515	0.9509	0.9508	0.9509
CNN1	0.8749	0.8706	0.8723	0.9482	0.9481	0.9406	0.9481

**Table 7:** Classification performance of CNN1 and CNN2 in HAR, with the SI strategy and 9 subjects on validation.

Model	Macro Average			Weighted Average			Accuracy
	Precision	Recall	F1-Score	Precision	Recall	F1-Score	
CNN2	0.8734	0.8632	0.8638	0.9289	0.9275	0.9274	0.9275
CNN1	0.8624	0.8603	0.8546	0.9259	0.9239	0.9238	0.9239

## Data Availability

The HAPT dataset can be found on https://archive.ics.uci.edu/ml/datasets/smartphone-based+recognition+of+human+activities+and+postural+transitions (accessed on 11 February 2023). The SHO dataset can be found in the author’s ResearchGate profile or https://www.researchgate.net/profile/Muhammad_Shoaib20/publication/266384007_Sensors_Activity_Recognition_DataSet/data/542e9d260cf277d58e8ec40c/Sensors-Activity-Recognition-DataSet-Shoaib.rar (accessed on 11 February 2023).
